# Characteristics of people with high visit‐to‐visit glycaemic variability in Type 2 diabetes

**DOI:** 10.1111/dme.13435

**Published:** 2017-08-17

**Authors:** J. D. Noyes, E. Soto‐Pedre, L. A. Donnelly, E. R. Pearson

**Affiliations:** ^1^ School of Medicine Ninewells Hospital and Medical School University of Dundee Dundee UK; ^2^ Division of Molecular & Clinical Medicine Ninewells Hospital and Medical School University of Dundee Dundee UK

## Abstract

**Aims:**

Increased visit‐to‐visit glycaemic variability is independently associated with adverse outcomes in Type 2 diabetes. Our aim was to identify the patient characteristics associated with raised visit‐to‐visit glycaemic variability in people with Type 2 diabetes.

**Methods:**

A case–control study was conducted to establish associations between HbA_1c_ variability and clinical covariates in 10 130 people with Type 2 diabetes. Variability was calculated by two metrics [sd and coefficient of variation (CV)] from a minimum of four HbA_1c_ readings obtained over a 4‐year period. High and low variability groups were defined as the top and bottom tertile of the sd or CV, and used in logistic regression analyses including a number of clinical and biochemical covariates. The analyses were stratified into low mean (< 53 mmol/mol; 7%) and high mean (≥ 53 mmol/mol; 7%) HbA_1c_ groups.

**Results:**

Findings were consistent across both HbA_1c_ groups and variability metrics. Treatment, independent of other factors, was the most strongly associated covariate for the risk of high HbA_1c_ variability. A six‐fold increased risk was observed in the low HbA_1c_ group, between the most and least intense treatment regimens (*P* < 0.001). Similar findings were present in the high HbA_1c_ group with a three‐fold increase in risk (*P* < 0.001). In addition, male gender, younger age, reduced HDL‐cholesterol and increased BMI were all found to be independently associated with raised visit‐to‐visit glycaemic variability.

**Conclusions:**

Intensive treatment resulting in low mean HbA_1c_ was associated with marked increase in HbA_1c_ variability. Irrespective of diabetes control, the greatest visit‐to‐visit variability was observed in young, insulin resistant men.

## What's new?

- Increased visit‐to‐visit HbA_1c_ variability has previously been associated with increased risk of adverse outcomes, including microvascular and macrovascular disease.
- We determined the patient characteristics associated with raised visit‐to‐visit glycaemic variability, independent of the mean HbA_1c_ level and established that these people with highly variable Type 2 diabetes have increased cardiovascular disease risk factors including male gender, raised BMI and reduced HDL‐cholesterol compared with those with low variability.
- People with Type 2 diabetes receiving greater intensity of treatment (e.g. insulin treatment or triple oral therapy) have greater visit‐to‐visit variability than those who are diet or monotherapy treated.

**What's new?**

- Increased visit‐to‐visit HbA_1c_ variability has previously been associated with increased risk of adverse outcomes, including microvascular and macrovascular disease.
- We determined the patient characteristics associated with raised visit‐to‐visit glycaemic variability, independent of the mean HbA_1c_ level and established that these people with highly variable Type 2 diabetes have increased cardiovascular disease risk factors including male gender, raised BMI and reduced HDL‐cholesterol compared with those with low variability.
- People with Type 2 diabetes receiving greater intensity of treatment (e.g. insulin treatment or triple oral therapy) have greater visit‐to‐visit variability than those who are diet or monotherapy treated.

## Introduction

Pivotal studies over the years have demonstrated the beneficial effects of lowering HbA_1c_ on both micro‐ and macrovascular complications in Type 2 diabetes 1, 2. However, on‐going debate exists as to whether other factors, such as glycaemic variability, play a contributory role in the adverse outcomes of diabetes.

Glycaemic variability is the measure of glycaemic fluctuations over a given time. Clinically, it is an umbrella term for two distinct measurements: intraday variability (short‐term) and visit‐to‐visit variability (long‐term). HbA_1c_ is most often used as the measure of glycaemia in the latter. Currently, no ‘gold standard’ metric exists to measure HbA_1c_ variability, however, it is most commonly expressed as either the standard deviation (sd) or coefficient of variation (CV) of the glycaemia measures.

Many studies in Type 2 diabetes cohorts have shown positive associations between raised visit‐to‐visit variability and adverse outcomes, independent of mean HbA_1c_ level. A recent meta‐analysis identified that renal disease, cardiovascular disease and mortality were all independently associated with raised HbA_1c_ variability 3. This analysis included 13 studies, the largest of which contained > 4000 participants 4. Research in the field of intraday variability has shown that certain patient features and clinical factors are associated with raised short‐term glycaemic variability 5, 6, 7, 8. However, we identified no similar studies in the field of long‐term glycaemic variability.

The detrimental consequences of high intraday variability have been mapped at a cellular level and are well documented 9, 10, 11, 12. The cellular effects of elevated HbA_1c_ variability are unknown, which gives rise to the possibility that two distinct biological processes are occurring. Establishing whether the same patient characteristics are associated with both raised intraday and HbA_1c_ variability is of interest because the findings could provide an insight into the biological processes responsible for increased HbA_1c_ variability and its associated adverse outcomes.

The aim of our research was to identify the patient characteristics associated with the risk of raised HbA_1c_ variability in a large Type 2 diabetes cohort.

## Participants and methods

### Study setting and design

A case–control study of HbA_1c_ variability was conducted in Tayside and Fife (Scotland, UK). Data were gathered from the Scottish Care Information‐Diabetes Collaboration (SCI‐DC); the electronic health record system used in Scotland for people with diabetes. We identified a source population of 13 285 individuals with Type 2 diabetes. Biochemical, demographic and prescribing data were available for these participants across a 20‐year period since 1994.

**Figure 1:**
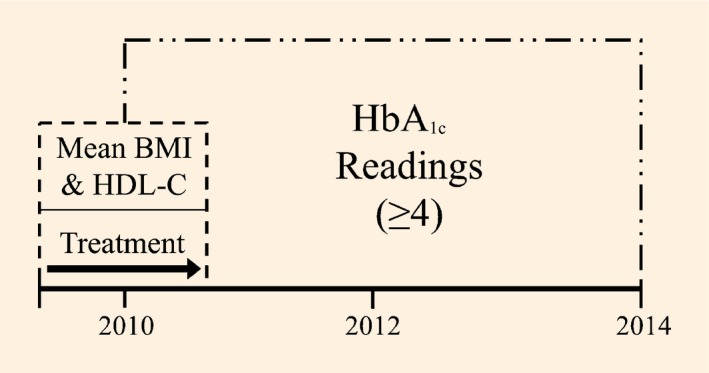
Depiction of the retrospective data collection process.

Participants with at least four HbA_1c_ recordings within a 4‐year window of time between 1 January 2010 and 1 January 2014 were included in this study (Fig.1). Baseline data on covariates were gathered at 1 January 2010 (± 6 months). If a participant had more than one covariate recording, the mean of these values was calculated. For those with more than one treatment therapy recorded, the latest listed was used for the analysis; this treatment was the most likely to be continued due to the stepwise progression of diabetes management.

**Figure 2:**
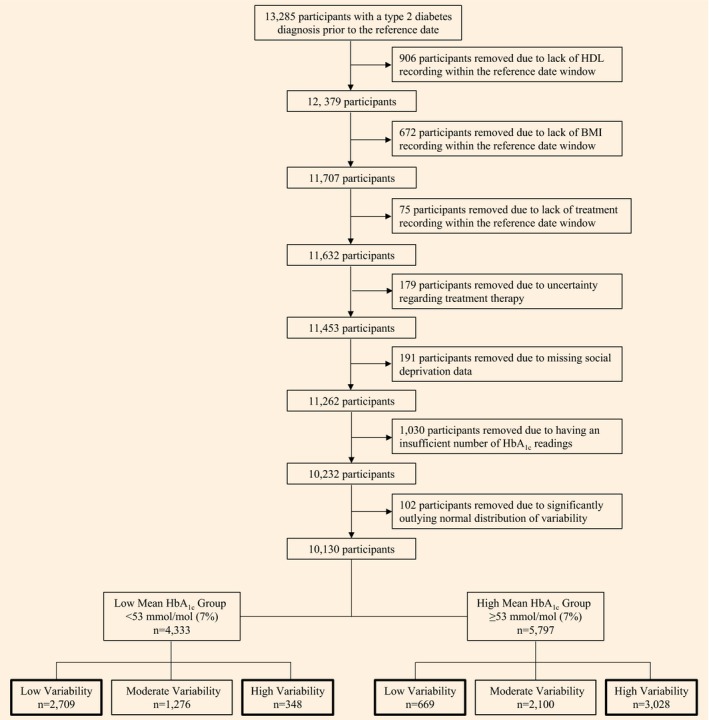
Flow chart showing how the participant sub‐groups were created when variability was defined as the standard deviation of the HbA_1c_ readings.

Each participant had their glycaemic variability defined by two metrics; the sd and the CV, which was 100 × sd/mean HbA_1c_. Two HbA_1c_ variability groups were then constructed encompassing the top tertile of the distribution (high variability = cases) and bottom tertile of the distribution (low variability = controls) respectively (Fig.2). This process was carried out for both sd and CV.

### Statistical analyses

Analysis of variance (ANOVA) and chi‐square tests were used to compare means and frequencies among subgroups of participants respectively. Tests of association with HbA_1c_ variability (coded as binary, high = case vs. low = control) were performed using unconditional logistic regression models. Univariate models were used to determine the patient characteristics associated with glycaemic variability, and potential determinants (gender, age, duration of Type 2 diabetes, diabetes treatment, HDL‐cholesterol, BMI, social deprivation and number of HbA_1c_ readings) were considered in the analysis. We then developed a multivariate model, including covariates where the univariate *P*‐value for the trait association was ≤ 0.2 13. This analysis was carried out for both variability metrics (i.e. sd and CV). We used the goodness‐of‐fit approach described by Hosmer and Lemeshow to test how well the derived model fitted the data 14. Two‐way interactions were additionally tested for between participant gender and the other covariates included in our final multivariate model.

Preliminary analyses revealed a strong positive association between mean HbA_1c_ level and high variability. Subsequently, the data were stratified into two groups based upon mean HbA_1c_ level. The HbA_1c_ value of 53 mmol/mol (7.0%) was selected as the cut‐off, creating a low mean HbA_1c_ group (< 53 mmol/mol; 7.0%) and a high mean HbA_1c_ group (≥ 53 mmol/mol; 7.0%) which were used in all subsequent analyses. This HbA_1c_ cut‐off of 53 mmol/mol (7.0%) was chosen for two reasons: first, it split our sample distribution into two groups with a large number of participants in each; and second, it is a clinical target for HbA_1c_ treatment in people with Type 2 diabetes 15.

Sensitivity analyses were performed to determine if extreme mean HbA_1c_ values were driving the analyses and whether insulin treatment, in isolation, was associated with increased variability. The first analysis removed all participants with a mean HbA_1c_ < 48 mmol/mol (6.5%) and > 75 mmol/mol (9.0%) from the cohort, after which the previously described multivariate analysis was carried out. The second sensitivity analysis was a whole sample multivariate analysis in which the three previously used treatment groups were broken down into five individual categories to specifically focus on any potential associations between the use of insulin and variability. The final sensitivity analysis carried out addressed the issue of therapy changes across the 4‐year HbA_1c_ collection window. Participants who had a different final treatment from their baseline recording were removed prior to the creation of the model. Statistical analyses were conducted using STATA/SE version 14 software (StataCorp, College Station, TX, USA), and the statistical significance level set at *P* < 0.05.

## Results

Some 10 130 participants were included in the analysis and their baseline characteristics are shown in Table1. A total of 3378 participants were categorised as having low HbA_1c_ variability, of whom 2709 had a mean HbA_1c_ < 53 mmol/mol (7.0%) and 669 had a mean HbA_1c_ ≥ 53 mmol/mol (7.0%). The high HbA_1c_ variability group contained 3376 participants, of whom 348 had a mean HbA_1c_ < 53 mmol/mol (7.0%) and 3028 had a mean HbA_1c_ ≥ 53 mmol/mol (7.0%). The characteristics of the 1030 people with Type 2 diabetes excluded from the analysis due to an insufficient number of HbA_1c_ readings are shown in Table S1.

**Table 1:** Baseline characteristics

Characteristic	All (*n* = 10 130)	Low variability controls (*n* = 3378)	High variability cases (*n* = 3376)	P value (low vs high)
Gender, *n* (%)				< 0.001
Female	4628 (45.7)	1735 (51.4)	1452 (43.0)	
Male	5502 (54.3)	1643 (48.6)	1924 (57.0)	
Age (years)	66.9 (11.1)	70.1 (10.4)	64.3 (11.4)	< 0.001
Type 2 diabetes duration (years)	5.1 (4.0)	4.5 (3.8)	5.6 (4.1)	< 0.001
Treatment, *n* (%)				< 0.001
Diet	3386 (33.4)	1805 (53.4)	638 (18.9)	
Mono or dual	5456 (54.9)	1456 (43.1%)	2008 (59.5)	
Triple or insulin	1288 (12.7)	117 (3.5%)	730 (21.6)	
HDL‐cholesterol (mmol/L)	1.22 (0.34)	1.32 (0.37)	1.15 (0.31)	< 0.001
BMI (kg/m^2^)	31.7 (6.2)	30.4 (5.9)	33.0 (6.5)	< 0.001
Social deprivation (SIMD), *n* (%)				< 0.001
1 (most deprived)	2110 (20.8)	667 (19.8)	764 (22.6)	
2	2189 (21.6)	703 (20.8%)	788 (23.3%)	
3	2006 (19.8)	627 (18.6%)	682 (20.2%)	
4	1923 (19.0)	679 (20.1%)	603 (17.9%)	
5 (least deprived)	1902 (18.8)	702 (20.8%)	539 (16.0%)	
No. of readings	7.9 (2.6)	7.0 (2.0)	8.7 (2.9)	< 0.001
HbA_1c_ (mmol/mol)	57 (12)	48 (7)	66 (12)	< 0.001
HbA_1c_ (%)	7.4 (1.1)	6.5 (0.7)	8.2 (1.1)	< 0.001

Values are reported as the mean (sd), unless indicated otherwise.

Similar results were observed using both the sd and CV as a measure of HbA_1c_ variability. However, the ‘goodness of fit’ score in the logistic regression models was superior when using the sd compared to the CV. Therefore, the results of the analysis are presented purely for the sd.

Univariate logistic regression revealed that a number of covariates were associated with HbA_1c_ variability in both the low and high mean HbA_1c_ strata (Table S2). The low mean HbA_1c_ analysis found that: men had greater odds of having high HbA_1c_ variability compared with women [ odds ratio (OR) 1.45, 95% CI 1.16–1.82, *P* = 0.001]; participants aged < 55 years were more likely to be highly variable compared with those aged 75 years and older (OR 3.05, 95% CI 2.12–4.39, *P* < 0.001); and both lower HDL‐cholesterol and higher BMI were associated with raised HbA_1c_ variability. Participants treated with medication had markedly increased variability compared with those whose diabetes was diet controlled. The odds of high HbA_1c_ variability were greater in the ‘triple or insulin’ group compared with the ‘mono or dual’ therapy group. Finally, a short duration of diabetes was marginally associated with lower risk of high HbA_1c_ variability. Similar results were seen in the high mean HbA_1c_ group, although the previously observed protective effect regarding duration of Type 2 diabetes was not significant in this analysis.

The results of the stratified multivariate logistic regression models are shown in Table2. The models were adjusted for social deprivation and the number of HbA_1c_ measures. These multivariate models are consistent with the univariate analysis showing that the previous findings are independent of other variables. The goodness‐of‐fit scores for the low (chi^2^ = 1736.72, *P* = 0.82) and high (chi^2^ = 2219.15, *P* = 0.72) mean HbA_1c_ strata models indicated that both fitted the data well. In addition, none of the two‐way interactions tested between gender and other covariates were statistically significant. Table 2 shows that those in the ‘triple or insulin’ treatment group were over six times more likely to be highly variable than those whose diabetes was diet treated in the low mean HbA_1c_ strata (OR = 6.64, 95% CI 3.72–11.86, *P* < 0.001). This large increase in risk was also observed in the high mean HbA_1c_ group with a greater than three‐fold increase compared with those whose diabetes was diet treated (OR 3.15, 95% CI 2.21–4.47, *P* < 0.001). In addition to this, younger people with Type 2 diabetes when compared with their older counterparts remained far more likely to be highly variable. In both HbA_1c_ strata those < 55 years old were more than twice as likely to have high HbA_1c_ variability compared to those aged 75 and older.

**Table 2:** Multivariate whole sample analysis showing the odds of being highly variable

Variable	Low mean HbA_1c_ (*n* = 3057)		High mean HbA_1c_ (*n* = 3697)	
	Odds ratio (95% CI)	*P*‐value	Odds ratio (95% CI)	*P*‐value
Gender				
Female	1.00		1.00	
Male	1.35 (1.05–1.74)	0.018	1.24 (1.03–1.49)	0.023
Age, years				
≥ 75	1.00		1.00	
≥ 65 to < 75	1.16 (0.86–1.58)	0.328	0.88 (0.70–1.11)	0.287
55 to < 65	1.22 (0.87–1.72)	0.249	1.49 (1.16–1.92)	0.002
< 55	2.29 (1.51–3.49)	< 0.001	2.36 (1.72–3.24)	< 0.001
Type 2 diabetes duration, years				
> 7	1.00		1.00	
2.5–7	0.86 (0.63–1.16)	0.322	0.95 (0.76–1.18)	0.618
< 2.5	0.98 (0.70–1.37)	0.904	1.36 (1.04–1.79)	0.026
Treatment				
Diet	1.00		1.00	
Mono or dual	3.02 (2.30–3.96)	<0.001	1.44 (1.14–1.82)	0.002
Triple or insulin	6.64 (3.72– 11.86)	<0.001	3.15 (2.21–4.47)	< 0.001
HDL‐cholesterol, mmol/L				
> 1.3	1.00		1.00	
1.0–1.3	1.47 (1.11–1.95)	0.007	1.39 (1.13–1.71)	0.002
< 1.0	1.79 (1.29–2.48)	< 0.001	1.87 (1.46–2.39)	< 0.001
BMI, kg/m^2^				
< 25	1.00		1.00	
25–35	1.15 (0.78–1.69)	0.479	1.22 (0.90–1.65)	0.205
> 35	1.62 (1.05–2.52)	0.030	1.72 (1.22–2.43)	0.002

Adjusted for social deprivation and number of readings.

Some 6763 participants were included in the first sensitivity analysis, which removed those with low mean HbA_1c_ (< 48 mmol/mol; 6.5%) and high mean HbA_1c_ (> 75 mmol/mol; 9%). The low variability group contained 2255 participants, of whom 1110 had a mean HbA_1c_ < 53 mmol/mol (7.0%) and 1145 had a mean HbA_1c_ ≥ 53 mmol/mol (7.0%). The high variability group in this analysis contained 2254 participants, of whom 225 had a mean HbA_1c_ < 53 mmol/mol (7.0%) and 2029 had a mean HbA_1c_ ≥ 53 mmol/mol (7.0%). This cohort was used in an identical multivariate model and showed that the same factors were associated with increased HbA_1c_ variability independent of other variables when extreme HbA_1c_ values are removed (Table S3). The goodness‐of‐fit scores for the low and high mean HbA_1c_ strata were (chi^2^ = 1014.87, *P* = 0.50) and (chi^2^ = 2058.30, *P* = 0.40) respectively.

Creating five individual treatment categories in the second sensitivity analysis revealed that those treated with insulin were more likely to be highly variable than those who were taking triple oral therapy in the low mean HbA_1c_ strata; however, this was not seen in the high mean HbA_1c_ group (Table S4). It is important to note that a larger number of participants were taking triple oral therapy or insulin in the high mean HbA_1c_ strata (*n* = 774) compared with the low mean HbA_1c_ strata (*n* = 73).

Of the 9804 participants with a documented final drug therapy, 6165 did not change their treatment therapy from baseline and were included in the third sensitivity analysis. The findings from this analysis were in keeping with the results of the previous multivariate analyses (Table S5). Further analysis revealed that 11.2% of participants in the low variability group changed their treatment during the 4 years compared with 58.8% of those in the high variability group.

## Discussion

The findings of our analysis revealed that young, insulin‐resistant men are most at risk of having high HbA_1c_ variability. In addition, the participant's prescribed treatment was found to be the largest independent predictor of risk. These findings were seen in both the low mean and high mean HbA_1c_ groups. The choice of variability metric, whether sd or CV, had little effect on the results of our analysis.

Comparing our results with the most similar study identified in the field of short‐term variability is of interest. Murata *et al*. 8 found in a cohort of 204 veterans with insulin‐treated Type 2 diabetes that high glucose variability over 8 weeks was associated with older age and a longer diabetes duration. In addition, obesity and those treated with larger insulin doses were found to have lower variability. By contrast, we show the opposite findings for HbA_1c_ variability. The difference between the patient characteristics associated with short‐term and long‐term variability implies that the underlying mechanisms responsible may differ. This discovery further supports the role of visit‐to‐visit variability as a discrete entity of the glycaemic variability research field and provides a starting point for future work to understand the biological mechanisms responsible for raised HbA_1c_ variability.

We show that the people with Type 2 diabetes most likely to have highly variable HbA_1c_ are those with high BMI and low HDL‐cholesterol. This is of interest given the previously reported association of HbA_1c_ variability with increased risk of adverse outcomes including cardiovascular disease 3, 4. Our findings suggest that this association may not be causally related to HbA_1c_ variability, but might reflect the high cardiovascular disease risk characteristics of this cohort.

HbA_1c_ variability is likely to be caused by a number of factors, predominantly related to variability in lifestyle, such as exercise 16, high stress 17 and poor treatment adherence 18. In addition to lifestyle factors, is it also likely that underlying biological mechanisms play a role in HbA_1c_ variability. For example, some people with Type 2 diabetes may be better than others at compensating for increased metabolic demands seen with episodic poor diet or illness. An extreme example of this can be found with glucokinase‐maturity‐onset diabetes of the young. These people with diabetes have impaired glucose‐sensing abilities which results in mildly elevated blood glucose levels 19. The HbA_1c_ readings of these individuals remain constant regardless of lifestyle and treatment interventions 20, consistent with these individuals being able to compensate for increased demand by increasing insulin secretion. The biological mechanisms underlying HbA_1c_ variability in Type 2 diabetes are not known, and further work is required in this area.

One potential limitation of our study was the positive correlation between mean HbA_1c_ level and HbA_1c_ variability. We wanted to draw associations based solely on HbA_1c_ variability, not mean HbA_1c_ level, which has previously proven associations with both micro‐ and macrovascular complications. To minimize this effect, we stratified the analysis by mean HbA_1c_ level. The results generated were consistent across both mean HbA_1c_ groups, strongly suggesting that the patient characteristics identified were associated with raised HbA_1c_ variability and not mean HbA_1c_ level. We also undertook a sensitivity analysis removing those with mean HbA_1c_ > 75 mmol/mol (9%) and < 48 mmol/mol (6.5%), and showed similar results to our full model. Another potential limitation to our model was underlying diabetes progression as those progressing rapidly will have a higher sd. To minimize the impact of progression we chose a 4‐year window for HbA_1c_ readings to accurately assess long‐term variability, while limiting the risk of falsely identifying fast progressors as having high HbA_1c_ variability. Finally, we acknowledge that this is an observational study, and although we have adjusted for potential measured confounders (such as social deprivation and number of HbA_1c_ measures) our results could still be affected by unmeasured confounders.

To determine HbA_1c_ variability, multiple HbA_1c_ readings are required. In this study, 1030 participants were excluded due to an insufficient number of HbA_1c_ measures, leaving a select group of people with Type 2 diabetes who have four or more HbA_1c_ readings over 4 years for further analysis. There are several potential clinical reasons why a person with Type 2 diabetes may need to be monitored more closely than another which must be considered when interpreting our results. In this study, the number of readings was adjusted for in all our multivariate models and the baseline characteristics of this excluded cohort were similar to those of the participants included in our final analysis (Table S1).

Some 3639 participants changed their treatment over the 4‐year window used to define HbA_1c_ variability, with treatment change being more common in the high variability group than the low variability group. This difference might reflect that those who are more variable are consequently more likely to receive additional treatment (as for a given mean HbA_1c_, their HbA_1c_ readings are more likely to go high than those with low variability and this may precipitate new treatment). An alternative explanation is that starting a new drug results in a large change in HbA_1c_ that makes the HbA_1c_ recorded during these 4 years more variable (i.e. the HbA_1c_ variability is not a measure of intrinsic variability but is secondary to treatment change). To ensure that this latter scenario was not driving our results, a sensitivity analysis was carried out which showed almost identical results to our main analysis.

The treatment prescribed to a person with Type 2 diabetes was found to be strongly associated with their risk of having highly variable HbA_1c_. This is particularly striking in the stratum with mean HbA_1c_ < 53 mmol/mol (7%). In this group, the participants who were intensively treated, with triple oral therapy or insulin, to achieve this target had a more than six‐fold increased risk of having highly variable HbA_1c_. Within this group, greater variability again was seen in those on insulin compared with triple oral therapy, although the numbers were small. A similar pattern is seen in the stratum with HbA_1c_ ≥ 53 mmol/mol (7%), although the effect is less strong and there is no greater risk of insulin treatment over triple oral therapy. These results are of interest, especially in the context of the outcomes of the ACCORD study where aggressive treatment initiated to achieve a treatment target of 48m mol/mol (6.5%) resulted in increased mortality compared with a less aggressive target 21. Our findings show that people with Type 2 diabetes who are treated intensively to reach a mean HbA_1c_ < 53 mmol/mol (7%) have much more variability between visits in their HbA_1c_ than those who are likely closer to diagnosis requiring no treatment or monotherapy. At this level, 11.5% of the participants with highly variable HbA_1c_ on triple oral therapy or insulin had at least one HbA_1c_ reading < 37 mmol/mol (5.5%); whereas 46.2% of the same cohort had a reading > 69 mmol/mol (8.5%). If HbA_1c_ variability per se is associated with increased risk of cardiovascular disease, this may explain the adverse outcomes found in ACCORD. Our findings support the rationale in the guidelines that emerged after ACCORD, that there should be a low HbA_1c_ target in those early in the disease process and a less aggressive target once treatment has intensified 15, 22. It should be noted, however, that our analysis cannot ascribe a causal association between intensive treatment regimens and high HbA_1c_ variability. Individuals with high HbA_1c_ variability will have large fluctuations in HbA_1c_ that are likely to result in treatment intensification; conversely more intense treatment, especially with insulin, may result in more variability in glycaemic control.

In conclusion, we report, for the first time, the patient characteristics associated with high and low visit‐to‐visit variability in HbA_1c_. We have shown that intensive treatment is associated with high HbA_1c_ variability, especially in those with a mean HbA_1c_ < 53 mmol/mol (7%). We also demonstrate that those with high HbA_1c_ variability are more likely to be male, younger and with low HDL‐cholesterol. Further work is required to investigate how HbA_1c_ variability varies over the life course of someone's diabetes and with different diabetes treatments; and more complex models are required to account for underling disease progression. Our findings suggest that those who are more likely to have high HbA_1c_ variability are those who have more cardiovascular risk factors. Given this, it is uncertain whether the adverse cardiovascular disease outcomes reported to be associated with increased HbA_1c_ variability can be attributed to the high HbA_1c_ variability per se, or more simply reflects the high cardiovascular risk factors seen in people with Type 2 diabetes who exhibit high HbA_1c_ variability. One potential way to unravel this causality question would be to use Mendelian randomization if suitable genetic instruments can be found.

## Funding sources

The study was supported by the Wellcome Trust.

## Competing interest

None declared.

## Supporting information


**Table S1.** Baseline characteristics of included and excluded participants.
**Table S2.** Univariate whole sample analysis showing the odds of high HbA_1c_ variability.
**Table S3.** Sensitivity analysis 1: multivariate analysis showing the odds of high HbA_1c_ variability with extreme mean HbA_1c_ values removed.
**Table S4.** Sensitivity analysis 2: multivariate whole sample analysis showing the odds of high HbA_1c_ variability with further differentiation of treatment groups.
**Table S5.** Sensitivity analysis 3: multivariate analysis showing the odds of high HbA_1c_ variability with participants that changed treatment therapy removed.

## References

[1] UK Prospective Study Group . Intensive blood‐glucose control with sulphonylureas or insulin compared with conventional treatment and risk of complications in patients with Type 2 diabetes (UKPDS 33). Lancet 1998; 352: 837–853. 9742976

[2] Holman RR , Paul SK , Bethel MA , Matthews DR , Neil HAW . 10‐year follow‐up of intensive glucose control in Type 2 diabetes. N Engl J Med 2008; 359: 1577–1589. 18784090 10.1056/NEJMoa0806470

[3] Gorst C , Kwok CS , Aslam S , Buchan I , Kontopantelis E , Myint PK *et al* Long‐term glycemic variability and risk of adverse outcomes: a systematic review and meta‐analysis. Diabetes Care 2015; 38: 2354–2369. 26604281 10.2337/dc15-1188

[4] Hirakawa Y , Arima H , Zoungas S , Ninomiya T , Cooper M , Hamet P *et al* Impact of visit‐to‐visit glycemic variability on the risks of macrovascular and microvascular events and all‐cause mortality in Type 2 diabetes: the ADVANCE trial. Diabetes Care 2014; 37: 2359–2365. 24812434 10.2337/dc14-0199

[5] Jin S‐M , Kim T‐H , Bae JC , Hur KY , Lee M‐S , Lee M‐K *et al* Clinical factors associated with absolute and relative measures of glycemic variability determined by continuous glucose monitoring: an analysis of 480 subjects. Diabetes Res Clin Pract 2014; 104: 266–272. 24630619 10.1016/j.diabres.2014.02.003

[6] Greven WL , Beulens JWJ , Biesma DH , Faiz S , de Valk HW . Glycemic variability in inadequately controlled type 1 diabetes and Type 2 diabetes on intensive insulin therapy: a cross‐sectional, observational study. Diabetes Technol Ther 2010; 12: 695–699. 20687867 10.1089/dia.2010.0044

[7] Kohnert K‐D , Augstein P . Glycemic variability correlates strongly with postprandial beta‐cell dysfunction in a segment of Type 2 diabetic patients using oral hypoglycemic agents. Diabetes Care 2009; 32: 1058–1062. 19244086 10.2337/dc08-1956 PMC2681045

[8] Murata GH , Duckworth WC , Shah JH , Wendel CS , Hoffman RM . Sources of glucose variability in insulin‐treated Type 2 diabetes: The Diabetes Outcomes in Veterans Study (DOVES). Clin Endocrinol (Oxf) 2004; 60: 451–456. 15049959 10.1111/j.1365-2265.2004.02001.x

[9] Risso A , Mercuri F , Quagliaro L , Damante G , Ceriello A . Intermittent high glucose enhances apoptosis in human umbilical vein endothelial cells in culture. Am J Physiol Metab 2001; 281: 924–930. 10.1152/ajpendo.2001.281.5.E924 11595647

[10] Monnier L , Mas E , Ginet C , Francoise M , Villon L , Cristol J *et al* Activation of oxidative stress by in patients with Type 2 diabetes. JAMA 2006; 295: 1681–1687. 16609090 10.1001/jama.295.14.1681

[11] Qu Y , Jacober SJ , Zhang Q , Wolka LL , DeVries JH . Rate of hypoglycemia in insulin‐treated patients with Type 2 diabetes can be predicted from glycemic variability data. Diabetes Technol Ther 2012; 14: 1008–1012. 23101951 10.1089/dia.2012.0099

[12] Razavi Nematollahi L , Kitabchi AE , Stentz FB , Wan JY , Larijani BA , Tehrani MM *et al* Proinflammatory cytokines in response to insulin‐induced hypoglycemic stress in healthy subjects. Metabolism 2009; 58: 443–448. 19303962 10.1016/j.metabol.2008.10.018

[13] Maldonado G , Greenland S . Simulation study of confounder‐selection strategies. Am J Epidemiol 1993; 138: 923–936. 8256780 10.1093/oxfordjournals.aje.a116813

[14] Lemeshow S , Hosmer W . A review of goodness of fit statistics for use in the development of logistic regression models. Am J Epidemiol 1982; 115: 92–106. 7055134 10.1093/oxfordjournals.aje.a113284

[15] Scottish Intercollegiate Network Guidelines (SIGN). Management of Diabetes. SIGN guideline 116. SIGN, Edinburgh, 2013.

[16] Støa EM , Meling S , Nyhus L‐K , Glenn Strømstad G , Mangerud KM , Helgerud J *et al* High‐intensity aerobic interval training improves aerobic fitness and HbA_1c_ among persons diagnosed with Type 2 diabetes. Eur J Appl Physiol 2017; 117: 455–467. 28160083 10.1007/s00421-017-3540-1

[17] Bralić Lang V , Bergman Marković B , Vrdoljak D . The association of lifestyle and stress with poor glycemic control in patients with diabetes mellitus Type 2: a Croatian nationwide primary care cross‐sectional study. Croat Med J 2015; 56: 357–365. 26321029 10.3325/cmj.2015.56.357 PMC4576750

[18] Osborn C , Mayberry L , Kim J . Medication adherence may be more important than other behaviours for optimizing glycaemic control among low‐income adults. J Clin Pharm Ther 2016; 41: 256–259. 26939721 10.1111/jcpt.12360 PMC4871756

[19] Chakera AJ , Steele AM , Gloyn AL , Shepherd MH , Shields B , Ellard S *et al* Recognition and management of individuals with hyperglycemia because of a heterozygous glucokinase mutation. Diabetes Care 2015; 38: 1383–1392. 26106223 10.2337/dc14-2769

[20] Stride A , Shields B , Gill‐Carey O , Chakera AJ , Colclough K , Ellard S *et al* Cross‐sectional and longitudinal studies suggest pharmacological treatment used in patients with glucokinase mutations does not alter glycaemia. Diabetologia 2014; 57: 54–56. 24092492 10.1007/s00125-013-3075-x PMC3855531

[21] The Action to Control Cardiovascular Risk in Diabetes Study Group . Effects of intensive glucose lowering in Type 2 diabetes. N Engl J Med 2008; 358: 2545–2559. 18539917 10.1056/NEJMoa0802743 PMC4551392

[22] National Institute for Health and Care Excellence (NICE) . Type 2 diabetes in adults: management. NICE guideline [NG 28]. NICE, London, 2015. 26741015

