# Supplementary material for: Characteristics of people with high visit‐to‐visit glycaemic variability in Type 2 diabetes
Source: Diabet Med. 2017 Aug 17;35(2):262–9. doi: 10.1111/dme.13435 (PMC5811920; doi:10.1111/dme.13435)
Supplement: Supplementary file 1 — Table S1. Baseline characteristics of included and excluded participants. Table S2. Univariate whole sample analysis showing the odds of high HbA1c variability. Table S3. Sensitivity analysis 1: multivariate analysis showing the odds of high HbA1c variability with extreme mean HbA1c values removed. Table S4. Sensitivity analysis 2: multivariate whole sample analysis showing the odds of high HbA1c variability with further differentiation of treatment groups. Table S5. Sensitivity analysis 3: multivariate analysis showing the odds of high HbA1c variability with participants that changed treatment therapy removed. [file DME-35-262-s001.docx]

**Characteristics of people with high visit-to-visit glycaemic variability in Type 2 diabetes**

J. D. Noyes^1^, E. Soto-Pedre^2^, L. A. Donnelly^2^ and E. R. Pearson^2^

^1^School of Medicine, and ^2^Division of Molecular & Clinical Medicine, Ninewells Hospital and Medical School, University of Dundee, Dundee, UK

**Supporting Information**

Table S1 Baseline characteristics of included and excluded participants

| Characteristic | Final Analysis Cohort  (n=10,130) | Excluded due to <4 HbA1c Measures  (n=1,030) |
| --- | --- | --- |
| Gender:  Female  Male | 4,628 (45.7%)  5,502 (54.3%) | 472 (45.8%)  558 (54.2%) |
| Age (years) | 66.9 (11.1) | 72.0 (13.5) |
| Type 2 Diabetes Duration (years) | 5.1 (4.0) | 5.4 (4.0) |
| Treatment:  Diet  Mono or Dual  Triple or Insulin | 3,386 (33.4%)  5,456 (54.9%)  1,288 (12.71%) | 424 (41.2%)  514 (49.9%)  92 (8.9%) |
| HDL-Cholesterol (mmol/l) | 1.22 (0.34) | 1.24 (0.38) |
| BMI (kg/m^2^) | 31.7 (6.2) | 29.9 (6.5) |
| Social Deprivation (SIMD)  1 (Most Deprived)  2  3  4  5 (Least Deprived) | 2,110 (20.8%)  2,189 (21.6%)  2,006 (19.8%)  1,923 (19.0%)  1,902 (18.8%) | 223 (21.7%)  254 (24.7%)  167 (16.2%)  205 (19.9%)  181 (17.6%) |
| Number of Readings | 7.9 (2.6) | 2.16 (0.8) |
| HbA1c (mmol/mol) | 57 (12) | 54 (16) |
| HbA1c (%) | 7.4 (1.1) | 7.1 (1.5) |

The values are reported as the mean (SD), unless otherwise indicated.

Table S2: Univariate whole sample analysis showing the odds of high HbA_1c_ variability

| Variable | Low Mean HbA1c (n=3,057) | | High Mean HbA1c (n=3,697) | |
| --- | --- | --- | --- | --- |
|  | Odds Ratio (95% CI) | P value | Odds Ratio (95% CI) | P value |
| Gender:  Female  Male | 1.00  1.45 (1.16-1.82) | 0.001 | 1.00  1.44 (1.22-1.70) | <0.001 |
| Age:  ≥75 years  ≥65- <75 years  ≥55- <65 years  <55 years | 1.00  1.43 (1.07-1.90)  1.60 (1.17-2.19)  3.05 (2.12-4.39) | 0.015  0.003  <0.001 | 1.00  1.17 (0.95-1.45)  2.16 (1.71-2.71)  3.44 (2.57-4.62) | 0.145  <0.001  <0.001 |
| Type 2 Diabetes Duration:  >7 years  2.5-7 years  <2.5 years | 1.00  0.68 (0.51-0.90)  0.61 (0.46-0.80) | 0.006 <0.001 | 1.00  0.79 (0.65-0.96)  1.02 (0.82-1.27) | 0.018  0.857 |
| Treatment:  Diet  Mono or Dual  Triple or Insulin | 1.00  3.52 (2.75-4.52)  9.07 (5.39-15.27) | <0.001  <0.001 | 1.00  1.43 (1.17-1.75)  3.38 (2.51-4.55) | <0.001  <0.001 |
| HDL-Cholesterol  >1.3 mmol/l  1.0-1.3 mmol/l  <1.0 mmol/l | 1.00  1.80 (1.39-2.35)  2.39 (1.78-3.22) | <0.001  <0.001 | 1.00  1.71 (1.41-2.07)  2.59 (2.07-3.25) | <0.001  <0.001 |
| BMI:  <25 kg/m^2^  25-35 kg/m^2^  >35 kg/m^2^ | 1.00  1.51 (1.04-2.18)  2.81 (1.89-4.19) | 0.028  <0.001 | 1.00  1.57 (1.18-2.08)  2.72 (1.97-3.75) | 0.002  <0.001 |

Table S3: Sensitivity analysis 1: multivariate analysis showing the odds of high HbA_1c_ variability with extreme mean HbA_1c_ values removed

| Variable | Low Mean HbA1c (n=1,335) | | High Mean HbA1c (n=3,174) | |
| --- | --- | --- | --- | --- |
|  | Odds Ratio (95% CI) | P value | Odds Ratio (95% CI) | P value |
| Gender:  Female  Male | 1.00  1.39 (1.01-1.92) | 0.046 | 1.00  1.16 (0.99-1.36) | 0.065 |
| Age:  ≥75 years  ≥65- <75 years  ≥55- <65 years  <55 years | 1.00  0.97 (0.66-1.42)  1.10 (0.71-1.71)  2.43 (1.40-4.22) | 0.861  0.656  0.022 | 1.00  0.80 (0.65-0.98)  1.16 (0.94-1.44)  1.65 (1.27-2.16) | 0.029  0.176  <0.001 |
| Type 2 Diabetes Duration:  >7 years  2.5-7 years  <2.5 years | 1.00  1.01 (0.68-1.48)  0.93 (0.59-1.46) | 0.978  0.752 | 1.00  1.00 (0.83-1.21)  1.35 (1.07-1.69) | 0.978  0.010 |
| Treatment:  Diet  Mono or Dual  Triple or Insulin | 1.00  1.78 (1.24-2.57)  3.78 (1.91-7.49) | 0.002  <0.001 | 1.00  1.15 (0.94-1.40)  1.64 (1.22-2.20) | 0.166  0.001 |
| HDL-Cholesterol  >1.3 mmol/l  1.0-1.3 mmol/l  <1.0 mmol/l | 1.00  1.09 (0.77-1.56)  1.45 (0.95-2.19) | 0.619  0.084 | 1.00  1.28 (1.07-1.54)  1.64 (1.33-2.03) | 0.007  <0.001 |
| BMI:  <25 kg/m^2^  25-35 kg/m^2^  >35 kg/m^2^ | 1.00  1.18 (0.71-1.95)  1.63 (0.92-2.87) | 0.515  0.092 | 1.00  1.32 (1.01-1.73)  1.76 (1.31-2.38) | 0.041  <0.001 |

Adjusted for social deprivation and number of readings.

Table S4: Sensitivity analysis 2: multivariate whole sample analysis showing the odds of high HbA_1c_ variability with further differentiation of treatment groups

| Variable | Low Mean HbA1c (n=3,057) | | High Mean HbA1c (n=3,697) | |
| --- | --- | --- | --- | --- |
|  | Odds Ratio (95% CI) | P value | Odds Ratio (95% CI) | P value |
| Gender:  Female  Male | 1.00  1.36 (1.05-1.74) | 0.018 | 1.00  1.26 (1.05-1.52) | 0.015 |
| Age:  ≥75 years  ≥65- <75 years  ≥55- <65 years  <55 years | 1.00  1.18 (0.87-1.60)  1.24 (0.88-1.76)  2.39 (1.56-3.64) | 0.295  0.213  <0.001 | 1.00  0.86 (0.68-1.08)  1.44 (1.12-1.86)  2.29 (1.66-3.15) | 0.204  0.005  <0.001 |
| Type 2 Diabetes Duration:  >7 years  2.5-7 years  <2.5 years | 1.00  0.95 (0.69-1.31)  1.13 (0.80-1.61) | 0.768  0.491 | 1.00  1.08 (0.86-1.35)  1.68 (1.27-2.23) | 0.521  <0.001 |
| Treatment:  Diet  Mono  Dual  Triple  Insulin | 1.00  2.65 (1.99-3.53)  4.68 (3.23-6.78)  5.19 (2.39-11.26)  10.60 (4.88-22.98) | <0.001  <0.001  <0.001  <0.001 | 1.00  1.18 (0.93-1.50)  2.30 (1.72-3.07)  3.70 (2.38-5.75)  3.53 (2.29- 5.44) | 0.173  <0.001  <0.001  <0.001 |
| HDL-Cholesterol  >1.3 mmol/l  1.0-1.3 mmol/l  <1.0 mmol/l | 1.00  1.46 (1.10-1.94)  1.76 (1.27-2.45) | 0.008  0.001 | 1.00  1.40 (1.14-1.73)  1.84 (1.44-2.36) | 0.001  <0.001 |
| BMI:  <25 kg/m^2^  25-35 kg/m^2^  >35 kg/m^2^ | 1.00  1.16 (0.78-1.70)  1.62 (1.04-2.51) | 0.462  0.033 | 1.00  1.24 (0.91-1.68)  1.75 (1.24-2.48) | 0.173  0.002 |

Adjusted for social deprivation and number of readings.

Table S5: Sensitivity analysis 3: multivariate analysis showing the odds of high HbA_1c_ variability with participants that changed treatment therapy removed

| Variable | Low Mean HbA1c (n=2,235) | | High Mean HbA1c (n=1,877) | |
| --- | --- | --- | --- | --- |
|  | Odds Ratio (95% CI) | P value | Odds Ratio (95% CI) | P value |
| Gender:  Female  Male | 1.00  1.27 (0.99-1.64) | 0.063 | 1.00  1.47 (1.07-2.03) | 0.018 |
| Age:  ≥75 years  ≥65- <75 years  ≥55- <65 years  <55 years | 1.00  1.28 (0.94-1.75)  1.54 (1.09-2.19)  2.46 (1.57-3.86) | 0.122  0.014 <0.001 | 1.00  0.99 (0.69-1.43)  1.49 (0.97-2.29)  2.94 (1.60-5.41) | 0.963  0.065  0.001 |
| Type 2 Diabetes Duration:  >7 years  2.5-7 years  <2.5 years | 1.00  0.96 (0.70-1.32)  1.03 (0.73-1.45) | 0.808  0.862 | 1.00  0.85 (0.60-1.20)  1.63 (1.01-2.64) | 0.347  0.045 |
| Treatment:  Diet  Mono or Dual  Triple or Insulin | 1.00  5.12 (3.88-6.75)  18.31 (9.58- 34.98) | <0.001  <0.001 | 1.00  2.48 (1.58-3.89)  8.67 (4.69-15.96) | <0.001  <0.001 |
| HDL-Cholesterol  >1.3 mmol/l  1.0-1.3 mmol/l  <1.0 mmol/l | 1.00  1.26 (0.95-1.67)  1.54 (1.10-2.16) | 0.106  0.011 | 1.00  1.45 (1.03-2.05)  2.00 (1.29-3.09) | 0.035  0.002 |
| BMI:  <25 kg/m^2^  25-35 kg/m^2^  >35 kg/m^2^ | 1.00  1.64 (1.09-2.47)  1.94 (1.22-3.11) | 0.018  0.005 | 1.00  1.08 (0.68-1.76)  1.96 (1.09-3.54) | 0.724  0.025 |

Adjusted for social deprivation and number of readings.
